# Novel Osteomyocutaneous Flap Model for Vascularized Composite Allotransplantation

**DOI:** 10.1016/j.jpra.2024.05.016

**Published:** 2024-06-12

**Authors:** David L. Tran, Michael F. Cassidy, Sachin R. Chinta, Alay R. Shah, Ren-Wen Huang, Eduardo D. Rodriguez, Daniel J. Ceradini

**Affiliations:** a Hansjörg Wyss Department of Plastic Surgery, New York University Langone Health, New York, New York; b Department of Plastic and Reconstructive Surgery, Chang Gung Memorial Hospital, Chang Gung University, Taiwan

**Keywords:** VCA, Vascularized composite allotransplantation, Transplant immunology

## Abstract

**Background:**

Vascularized composite allotransplantation (VCA) has become a viable option for restoration of devastating injuries that are not amenable to conventional reconstructive techniques. However, the relative scarcity of procedures performed worldwide, as well as the potential for iatrogenic injury with biopsies, makes studying the immunopathogenesis of acute rejection challenging. Translational VCA research focuses on developing strategies to overcome these barriers with the use of animal models can be technically challenging and difficult to replicate without highly trained microsurgeons.

**Methods:**

We describe a modified model of a femur-based composite tissue allograft using an adapted vascular cuff anastomotic technique with a tunneled skin flap in a rodent model.

**Results:**

The use of a heterotopic osteomyocutaneous flap with a subcutaneously tunneled-skin paddle to the posterolateral aspect of the recipient rodent allows for ease of flap monitoring and reduces the risk of self-mutilation. A total of six transplantations were conducted with no signs of self-mutilation. Operative time decreased as our surgical technique improved, and long-term graft tolerance was possible under our immunosuppressive regimen. Additionally, we demonstrate cases of successful transplantation in both an allogeneic and syngeneic rodent model.

**Conclusion:**

Animal models, although technically challenging, are a reliable and reproducible modality that has been used to investigate various aspects of VCA immunology. We describe the success of an osteomyocutaneous flap with a modified vascular cuff anastomosis that can be used by investigators with less experience in microsurgical techniques to further our understanding of VCA physiology. Furthermore, tunneling of the skin paddle reduces the risk of self-mutilation and other external factors affecting the graft.

## Introduction

Vascularized composite allotransplantation (VCA) is a surgical procedure that involves the transplantation of multiple tissues, such as skin, muscle, bone, and nerve, as a functional unit from a donor to a recipient. VCA offers the potential to restore devastating tissue defects and injuries that cannot be treated by conventional reconstructive surgery. However, VCA also poses significant immunological challenges, as the transplanted tissues express a high degree of histocompatibility antigens, leading to the activation of the recipient's immune system and the potential for transplant rejection.

As a result, VCA research focuses on developing strategies to overcome these immunological barriers and improve transplant outcomes. One such strategy is the use of translational animal models, particularly small animals such as rodents, for studying VCA transplant rejection and immunology in a reproducible way. These animal models allow researchers to study the complex interactions between the transplanted tissues and the recipient's immune system, as well as to test the efficacy of new immunosuppressive regimens. Small animal models for VCA research allow for high reproducibility and ease of manipulation. Rodents are relatively small, inexpensive, and have a short lifespan, making them ideal for large-scale studies and longitudinal experiments. Despite these advantages, there are also limitations to using small animal models for VCA research, as they can be technically challenging to investigators with limited microsurgical experience or training.

By far, the most common rodent model of VCA utilizes a hind limb flap. The first and most rudimentary of such procedures was described by Harashina and Buncke, who performed an *autogenic orthotopic* hind limb amputation at the midfemoral shaft with replantation using an intramedullary nail and microvascular anastomosis.^1^ The first *allogeneic orthotopic* hind limb transplant was performed by Doi et al., which included both microvascular anastomoses and sciatic nerve coaptation.^2^ It was not until 2001, although, that Liao et al. pioneered the *modified heterotopic* hind limb transplant, describing the first osteomyocutaneous (OMC) flap model. In this model, the flap was anchored to the recipient gracilis, and skin paddle was placed on the medial thigh. This technique reduced operative time and morbidity and was primed for immunologic studies rather than graft function, which was emphasized by previous studies*.*^3^

Despite the development of highly analogous rodent models of VCA, these procedures require a multitude of resources, including expert technical skills, lengthy operative time, and prevention of recipient self-mutilation. In particular, an anastomosis failure rate of 1%-10% in human free tissue transfers has sparked research into alternatives to suture anastomosis, such as couplers, clips, adhesives, and even lasers.^4^, ^5^, ^6^, ^7^ In the present study, we proposed solutions to these challenges that we have implemented in our modified partial heterotopic hind limb transplant model. Specifically, we described our experience using an adapted microvascular anastomosis coupling technique and positioning of the OMC flap components.

## Methods

### Study Design

Four syngeneic and two allogenic transplantations were conducted using the modified cuff anastomotic technique. The syngeneic transplant consisted of a Lewis donor rat to a Lewis recipient to demonstrate technical success of the procedure without possible confounding from acute rejection. The allogeneic transplant consisted of a Brown Norway donor to a Lewis recipient. Rodents were administered subcutaneous heparin and buprenorphine daily on postoperative day (POD)0 to POD5. Allogeneic transplant recipients additionally received subcutaneous tacrolimus indefinitely until an experimental endpoint was reached where rejection was desired.

### OMC Flap Anatomy

**Figure 1:**
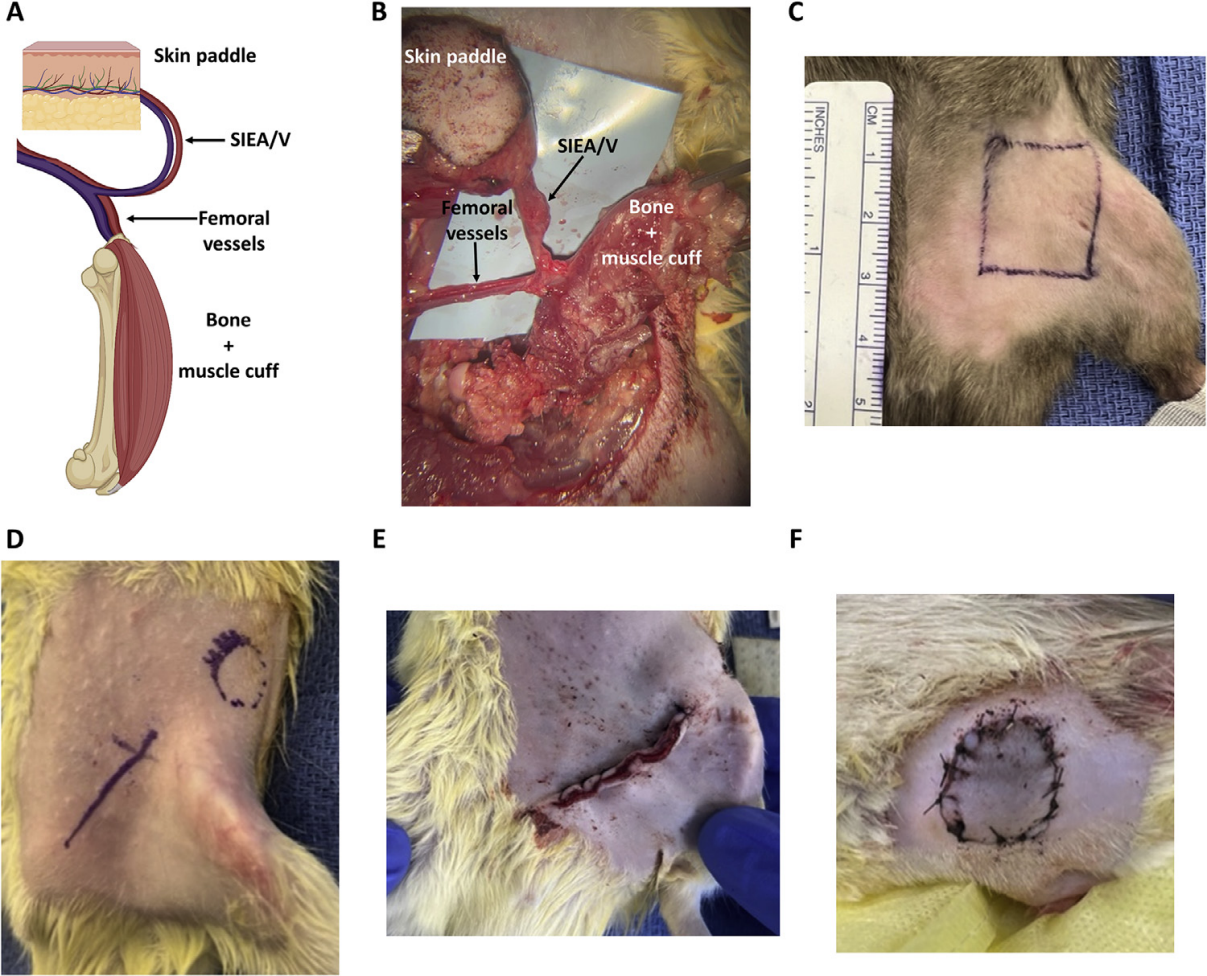
Flap Anatomy and Surgical Markings. (**A**) Diagram and (**B**) intraoperative image of osteomyocutaneous flap anatomy. Surgical markings for (**C**) donor and (**D**) recipient. Recipient closure of (**E**) groin and (**F**) skin paddle.

The pedicle of our OMC flap contains the femoral vessels. Diverging from these are the superficial inferior epigastric artery and vein (SIEA/V), which supply the skin paddle for cutaneous monitoring. The distal femoral vessels and subsequent branches supply the entire femur and anterior muscle compartment. These components are outlined in Figure 1A.

### Donor Surgical Technique

Following isoflurane induction, the donor groin was shaved, and a 2.5 × 2.5–cm square was marked on the skin (Figure 1C). The skin was cleaned with 70% isopropyl alcohol, and a superficial incision was started in the inferolateral corner of the marking using Stevens curved tenotomy scissors. Blunt dissection proceeded in the subcutaneous plane along the SIEA/V to their origin. The inguinal ligament was divided just cranial to the femoral vessels, and the skin and rectus muscle were retracted. The femoral vessels were carefully dissected to the adventitia, beginning at the inguinal ligament and proceeding distally. Murphy's branch of the common femoral vein and any branches of the femoral artery were suture ligated and divided to provide mobilization. Distally, the saphenous vessels were suture ligated and cauterized with bipolar forceps.

The femur was then disarticulated at the knee joint using bipolar forceps, and dissection proceeded proximally toward the hip joint along the posterior and lateral aspects of the bone. Muscles of the posterior and lateral thigh compartments were cauterized off the femur, taking care to preserve the periosteum. The popliteal vessels and sciatic nerve were divided using bipolar forceps. A muscle cuff from the anterior compartment was preserved, acting as the muscular component of the OMC flap. The femur was then disarticulated at the hip joint, with its only remaining attachment being the vascular pedicle (Figure 1B). The femoral vessels were clamped proximally with Rizzuti clips, cut with microscissors, and flushed with heparinized saline. The donor vein was everted over a polyamide cuff. Internal diameter was .71 mm for the vein cuff, whereas .82 mm was the external diameter of the medical tubing.

### Recipient Surgical Technique

Following identical induction and preparation, a 3-cm oblique incision was marked on the ipsilateral recipient groin, and the approximate position of the skin paddle was marked on the ipsilateral flank (Figure 1D). The oblique incision was dissected down to the femoral vessels, ensuring that the SIEA/V were inferior to the incision. Blunt dissection continued laterally toward the estimated skin paddle position, and the overlying skin was excised. Rectus muscle and the surrounding skin were retracted, and the femoral vessels were dissected in a similar fashion to the donor, ligating and dividing Murphy's branch and pertinent arterial branches. The femoral vessels were clamped just distal to the inguinal ligament and ligated just proximal to the SIEA/V. The vessels were then divided, and the recipient artery was everted over a polyamide cuff. The internal diameter was.58 mm, and the external diameter was .74 mm for the arterial cuff. Just prior to anastomosis, the muscle cuff was anchored to the rectus muscle, and the skin paddle was tunneled to the recipient flank, carefully avoiding inadvertent rotation of the vascular pedicle. Following anastomosis, the groin incision and skin paddle were closed with 4-0 nylon sutures in horizontal mattress and simple interrupted fashions, respectively, excising any excess tissue in the process (Figures 1E-F).

### Anastomosis Technique

**Figure 2:**
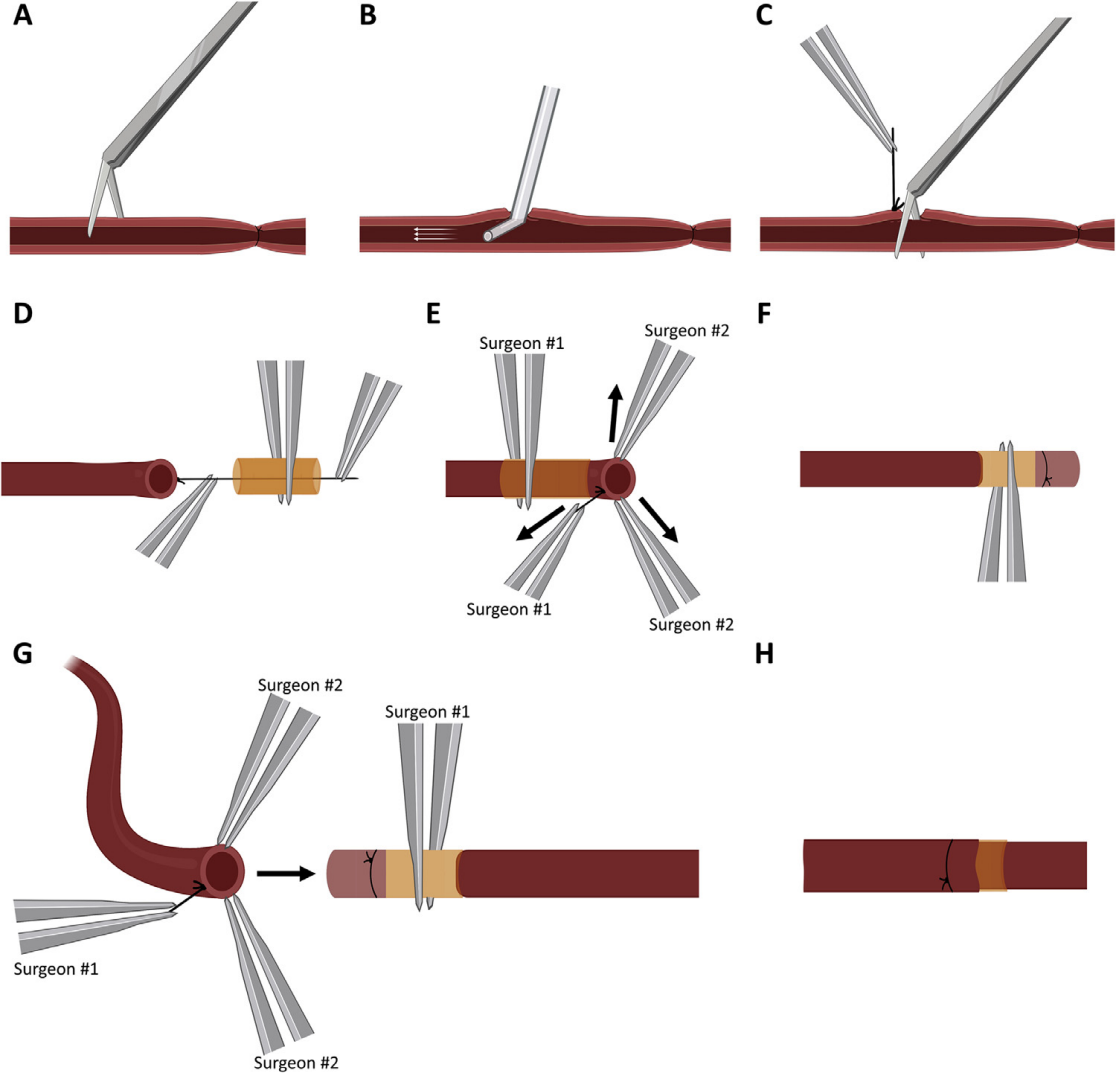
Vessel Anastomosis Steps. (**A**) Incomplete circumferential cut of the vessel with (**B**) heparinized saline flush. (**C**) Complete vessel transection following placement of stay suture. (**D**) Stay suture tail pulled through cuff and (**E**) everted over. (**F**) Everted vessel cinched in place. (**G**) Approximation of complement vessel over cuff using stay suture. (**H**) Complete cuff anastomosis. Created with BioRender.com

The identical technique was used for artery and vein anastomosis. Following ligation, an incomplete circumferential cut was performed on the vessel, such that access to the lumen was achieved while a point of attachment was preserved (Figure 2A). The vessel was then dilated and flushed with heparinized saline (Figure 2B). A single 9-0 stay suture was placed on the anastomosis side of the vessel, leaving a tail length of ∼6-8 mm. The vessel was then completely transected (Figure 2C). The stay suture tail was inserted into a precut 4-mm polyamide cuff and pulled through (Figure 2D). The cuff and stay suture were held by Surgeon #1, while Surgeon #2 held two equidistant points of the vessel (Figure 2E). Outward radial tension was gently applied at these three points, and the vessel was everted over the cuff in a parachute-like manner. While Surgeon #1 continued to hold the cuff and everted vessel in place, Surgeon #2 cinched the everted vessel with a 9-0 suture. The original stay suture was then excised, completing the cuffing (Figure 2F).

The complement vessel was approximated to the cuff, spread in a similar parachute-like manner using a stay suture, and enveloped the cuff. Surgeon #1 held the cuff and stay suture tail, while Surgeon #2 held two points on the vessel (Figure 2G). Surgeon #1 held the cuff and stay suture, maintaining oppositional tension on each, while Surgeon #2 cinched the outer vessel with a 9-0 suture. The stay suture was excised once again, completing the anastomosis (Figure 2H).

## Results

Six partial hind limb OMC flap transplantations were performed in this study. All rodents tolerated the procedure well and were independently ambulatory following recovery from anesthesia, demonstrating no evidence of distress, pain, or inadvertent neurovascular damage. Self-mutilation was not observed in this transplantation model due to the location of the tunneled-skin flap. Similarly, groin incisions healed well with no signs of self-mutilation. The initial procedures consisted of syngeneic transplantations and had a mean operative time of 315 ± 15 minutes. After the initial learning curve and stabilization of the operative technique, the mean operative time for our allogenic transplantations was 205 ± 2.5 minutes. Ischemia time (time to reperfusion of the OMC partial hind limb flap) was 204 ± 4 minutes in our initial syngeneic transplantations and 61 ± 15 minutes in the subsequent allogeneic transplantations. Flap viability was monitored daily via the flank skin paddle, and hair growth was observed in both cases (Supplementary Figure 1). Long-term allograft survival (>25 days) was seen under our postoperative protocol.

## Discussion

Small animal models such as rodents allow for high reproducibility and subsequent large-scale and longitudinal hypothesis testing, especially in VCA research. However, these models can be technically challenging to investigators with limited microsurgical experience. Additionally, rodent behavior can be unpredictable, and surgical sites are often irritating to the animals, leading to incidents of self-mutilation that can interfere with flap monitoring and experimental results.^8^^,^^9^ Using a model that allows for efficient transplantation of allografts with limited ischemia time and subsequent ease of monitoring provides a valuable means to study VCA physiology and rejection.

We address these issues by describing a rodent model with a subcutaneously tunneled skin paddle supplied by the SIEA to the recipient animal's flank. In our experience, placing the skin paddle in a remote area has incurred no episodes of self-mutilation and facilitates ease of daily monitoring. Additionally, external trauma to this skin paddle is theoretically reduced compared with traditional inset in the abdominal/groin skin, which can be inadvertently dragged along cage contents by the animal. Furthermore, dorsolateral placement of the skin paddle allows graft failure to be easily visualized without extensive manipulation of the rodent. Changes in skin quality with arterial and venous insufficiency are demonstrated by pallor and paddle congestion, respectively. In the setting of experimental models that look to assess rejection, erythematous changes, along with lack of hair growth and irritation, will be evident in allogeneic models that do not have adequate immunosuppression.

Our model disarticulates the femur at the hip and knee joints, obviating the need for a midfemoral osteotomy, as previously described in several other hind limb models.^10^^,^^11^ Doing so allows for transplantation of a large, vascularized bone marrow compartment without the associated morbidity or technical burden of multiple osteotomies. Furthermore, forgoing osteotomy limits flap ischemia and allows for local and systemic immunomodulation from the marrow compartment. Recent studies by Lin et al.^12^ have demonstrated that vascularized bone marrow transplantation could lead to immunomodulatory and protolerogenic effects that can potentially lead to a reduction in immunosuppressive requirements. This model offers a technically simpler method that can be used to develop future experiments for the analysis of full-compartment vascularized bone marrow transplantation.^13^

The most technically challenging portion of the procedure tends to be vessel preparation and vascular anastomosis. This is especially true for those who are otherwise inexperienced with microsurgical techniques. Novice investigators who are unfamiliar with manipulating vessels and performing anastomoses risk vessel injury with vascular complications, extended graft ischemia time, prolonged anesthesia times, and animal death, ultimately compromising experimental results. Our nonsuture vascular anastomosis aimed to address these concerns and is adapted from previously described cuff techniques that have been used for VCA research.^10^^,^^14^ Beginning with vessel preparation, performing an incomplete circumferential cut allows ease of manipulation while dilating and flushing of the flap compared with a freely mobile vessel. The single stay suture allows for streamlining of vascular cuffing while only requiring a small amount of time to place. Importantly, the stay suture tail length must exceed that of the cuff, such that it can be easily inserted and pulled through. Moreover, the stay suture makes vessel eversion and enveloping of the complement vessel substantially less cumbersome; the instrument grabbing the suture can be placed further away from the vessel, effectively creating more space for the other two instruments. Additionally, we employ the vascular cuff on not only the venous system, but the arterial system as well. We have noted this technique to be stable in the higher-pressure arterial system throughout the duration of the rodent's postoperative course. In theory, our vessel eversion and anastomotic cuffing technique can be performed by a single surgeon in which the cuff is clamped and stabilized with a single Rizzuti clip while the surgeon everts or approximates the complement vessel unassisted. Of note, previously described methods of nonsuture vascular anastomosis were commonly focused on mice populations as opposed to the rat model that was utilized in this study.^15^ Utilization of a rat model allows for application of microsurgical techniques, as opposed to super-microsurgical techniques, which can lower the skill that is required to carry out these complicated procedures.

In human free flap reconstructions, arterial anastomotic coupling has struggled to gain traction despite an 8% incidence of troubleshooting or failure.^16^ Nevertheless, cuff-based anastomosis is suitable for animal models in which operative time is shorter, and the consequences of failure are less severe. In our adaptation, use of a suture anchor and a second surgeon helps to make the technique faster and enhances the ease of vessel manipulation.

## Limitations

The primary limitation of this study is the modest sample size. Although advantageous for consistency, the smaller sample size potentially limits the external validity and translational applicability of our findings. Furthermore, although our model limits the requirement of technical skill, inherent variability in the performance of VCA by different practitioners could introduce an element of inconsistency, potentially affecting the reproducibility of our results across varying levels of microsurgical expertise. Additionally, the duration of our postoperative observations may not be sufficient to detect late-phase immunological events, such as chronic rejection, which could have significant implications for long-term graft survival and patient outcomes in clinical practice.

## Conclusions

As VCA has demonstrated technical success and feasibility in several instances, continued research focuses on understanding the mechanisms of rejection and tolerance in recipients. Animal models, although technically challenging, are a reliable and reproducible modality that has been used to investigate various aspects of VCA immunology. Taken together, we describe a modified heterotopic OMC flap with an adapted vascular cuff anastomosis that can be used by investigators less experienced with microsurgical techniques to further our understanding of VCA physiology. This technique obviates the need for femur osteotomy, utilizes both venous and arterial anastomotic coupling, and involves tunneling of a donor skin paddle to allow for efficient flap monitoring while also limiting the risk of self-mutilation. Overall, this model offers a technically simpler VCA model that can be used to develop future experiments for further study.

## Appendix Supplementary materials

Figure 1. Flap Viability Monitored via Flank Skin Paddle. (**A**) Flank skin paddle immediately following transplant operation. Initial dark discoloration can be attributed to intraoperative ischemia time. Flank skin paddle on (**B**) POD 2, (**C**) POD 3, and (**D**) POD 18. Skin color and hair growth act as surrogate markers for tissue perfusion and viability throughout the monitoring process Image, application 1
