# Supplementary figures and images for: Novel Osteomyocutaneous Flap Model for Vascularized Composite Allotransplantation
Source: JPRAS Open. 2024 Jun 12;41:244–51. doi: 10.1016/j.jpra.2024.05.016 (PMC11295284; doi:10.1016/j.jpra.2024.05.016)

**A**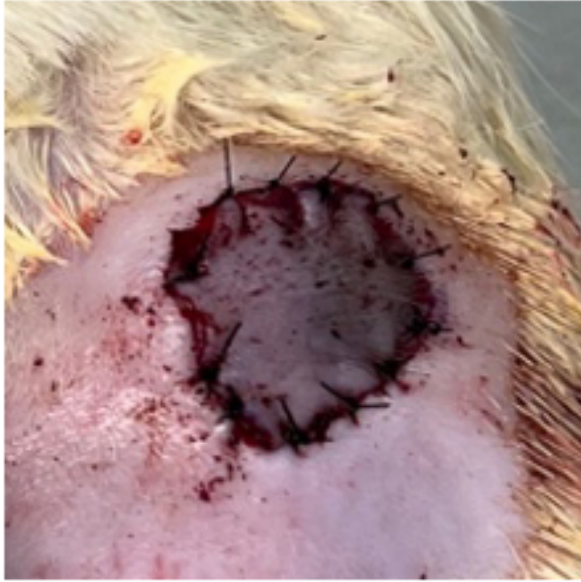**B**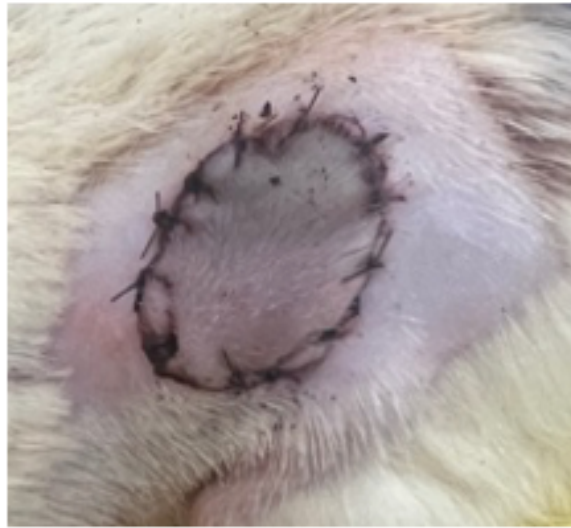**C**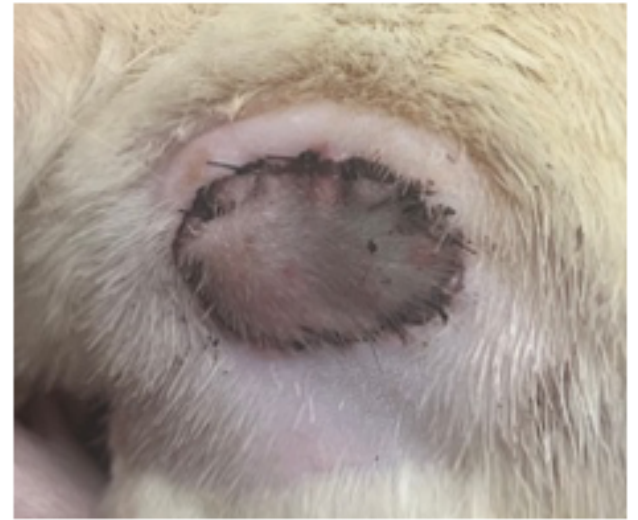**D**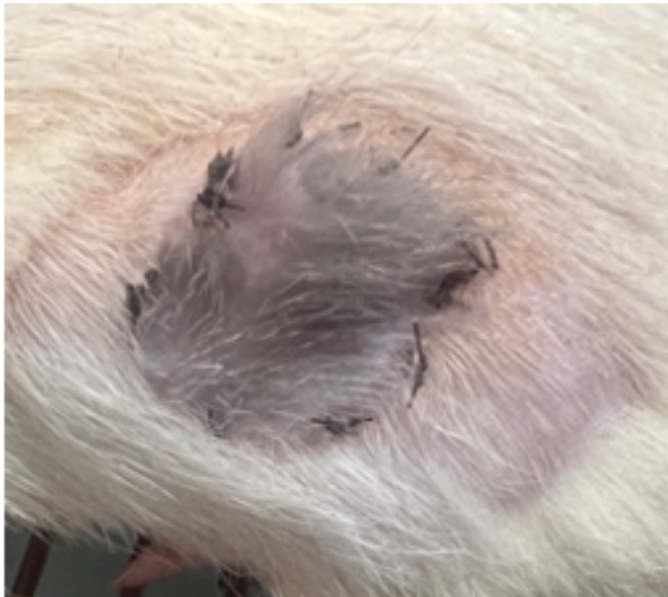

Supplement: Supplementary file 1 — Figure 1. Flap Viability Monitored via Flank Skin Paddle. (A) Flank skin paddle immediately following transplant operation. Initial dark discoloration can be attributed to intraoperative ischemia time. Flank skin paddle on (B) POD 2, (C) POD 3, and (D) POD 18. Skin color and hair growth act as surrogate markers for tissue perfusion and viability throughout the monitoring process [file mmc1.pdf]
